# Dutch women with a low birth weight have an increased risk of myocardial infarction later in life: a case control study

**DOI:** 10.1186/1742-4755-2-1

**Published:** 2005-01-10

**Authors:** Bea C Tanis, Kitty Kapiteijn, Ronella M Hage, Frits R Rosendaal, Frans M Helmerhorst

**Affiliations:** 1 Department of Hematology, Leiden University Medical Center, Leiden, The Netherlands; 2 Department of Gynecology and Reproductive Medicine, Leiden University Medical Center, The Netherlands; 3 Department of Clinical Epidemiology, Leiden University Medical Center, The Netherlands

## Abstract

**Background:**

To investigate whether low birth weight increases the risk of myocardial infarction later in life in women.

**Methods:**

Nationwide population-based case-control study. Patients and controls: 152 patients with a first myocardial infarction before the age of 50 years in the Netherlands. 568 control women who had not had a myocardial infarction stratified for age, calendar year of the index event, and area of residence.

**Results:**

Birth weight in the patient group was significantly lower than in control women (3214 vs. 3370 gram, mean difference -156.3 gram (95%CI -9.5 to -303.1). The odds ratio for myocardial infarction, associated with a birth weight lower than 3000 gram (20^th ^percentile in controls) compared to higher than 3000 gram was 1.7 (95%CI 1.1–2.7), while the odds ratio for myocardial infarction for children with a low birth weight (< 2000 g) compared to a birth weight ≥ 2000 g was 2.4 (95%CI 1.0 – 5.8). Both figures did not change after adjustment for putative confounders (age, education level, body mass index, waist-hip ratio, hypertension, diabetes, hypercholesterolemia, smoking, and family history of cardiovascular disease).

**Conclusions:**

Low birth weight is associated with an increased risk of myocardial infarction before age of 50 in Dutch women.

## Background

Intrauterine malnutrition, as reflected by birth weight and abnormal thinness at birth, has been associated with an increased incidence of risk factors for arterial disease, i.e. hypertension, impaired glucose tolerance, diabetes and to a lesser extent hyperlipidemia and body fat distribution in adulthood [1-10]. This observation has become known as the 'fetal origins of adult disease' or 'Barker hypothesis', which suggests that several of the major diseases of later life, including coronary heart disease, stroke and cardiovascular death, originate in impaired intrauterine growth and development [11,12]. In cohort studies, Barker [13-15] in England and Finland, Rich-Edwards *et al*. [16] as part of the Nurses' Health Study in the USA and Leon *et al. *[17] from Uppsala in Sweden showed an inverse relationship between birth weight and the clinical endpoint ischemic heart disease. Leon *et al. *[17] found a significant relationship only among male singletons and adjusted their results for gestational age and socioeconomic confounding. The association was not found in a cohort study from Gothenburg [18].

Our aim was to investigate the association in a case control study among Dutch women.

## Methods

The RATIO (Risk of Arterial Thrombosis In relation to Oral contraceptives) study is a population-based case-control study on myocardial infarction in relation to oral contraceptive use among women aged 18 to 49 years in the Netherlands [19]. An additional standardized questionnaire was sent to all 218 patients and 769 controls from whom also blood samples had been taken for determination of metabolic risk factors (diabetes and hypercholesterolemia). Questions elicited information on birth weight, waist and hip circumference and data on the menstrual cycle. For 13 women no current address could be found (12 patients, 1 control). Four women had died since the index date (2 patients, 2 controls), which was the date of the first myocardial infarction for the patients and the midyear for the controls. One hundred and fifty two patients (71%) and 568 controls (75%) responded to the questionnaire and women were asked to measure their waist and hip circumference. Body mass index (BMI) was calculated as body weight (kg) divided by height squared (m^2^). Waist-hip-ratio was calculated as waist circumference divided by hip circumference.

Multiple linear and unconditional logistic regression were used to analyze the data. Odds ratios for the relationship between birth weight and myocardial infarction were calculated and 95% confidence intervals (95%CI) were derived from the models. Birth weights were categorized according to quintiles in control women in order to investigate an association between birth weight and the risk of myocardial infarction later in life. These were <3000 g, 3000 to 3199 g, 3200 to 3499 g, 3500 to 3883 g, and >3884 g, respectively. To determine whether women with a lower birth weight had a higher risk for a myocardial infarction, patients were divided in a group with a birth weight equally or higher than 2000 g and a group with a birth weight lower 2000 g [20]. Odds ratios were adjusted for age, education level, body mass index, waist-hip ratio, hypertension, diabetes, hypercholesterolemia, smoking, and family history of cardiovascular disease, when appropriate. Interaction between low birth weight and low education level was investigated by computing a dummy variable.

## Results

The characteristics of 152 women with myocardial infarction and 568 control women at the index date are shown in Table 1. At the moment of completing the questionnaire, patients were aged 32–59 years (mean 50), and control women 25–60 years (mean 47). The mean body mass index was 25.1 kg/m^2 ^for the patients and 23.4 kg/m^2 ^for control women, mean difference 1.76 kg/m^2 ^(95%CI 1.05–2.47), p < 0.001. Ninety-seven patients (64%) and 415 (73%) controls could give their birth weight. Compared with control women, patients had a significantly lower mean birth weight (3214 vs. 3370 g, mean difference -156.3 g (95%CI -9.5 to -303.1). The odds ratio for myocardial infarction for children with a low birth weight (< 2000 g) compared to a birth weight ≥ 2000 g was 2.4 (95%CI 1.0 to 5.8). After adjustment for putative confounders (age, education level, body mass index, waist-hip ratio, hypertension, diabetes, hypercholesterolemia, smoking, and family history of cardiovascular disease) the odds ratio did not change. Odds ratios for myocardial infarction in different categories of birth weight as compared to the reference category (birth weight higher than 3884 g) were 1.3 (95%CI 0.5–3.3) for a birth weight 3500 to 3883 g, 1.4 (95%CI 0.6–3.4) for a birth weight 3200 to 3499 g, 1.7 (95%CI 0.6–5.1) for a birth weight 3000 to 3199 g, and 2.3 (95%CI 1.0–5.4) for a birth weight lower than 3000 g (Table 2). The risk of myocardial infarction was 6.2 fold increased (95%CI 2.7–13.9) among women with low birth weight and a low educational level compared to women with a high birth weight and a high educational level (reference category).

**Table 1:** Characteristics of patients with a first myocardial infarction and control women

**Characteristic**	**Patients (N = 152)**	**Control women (N = 568)**
Age – yr (SD)	42.1 (0.5)	38.6 (0.3)
Caucasian ethnicity (%)	142 (93)	538 (95)
Educational level		
- Primary school or less (%)	83 (55)	160 (28)
- Secondary school (%)	52 (34)	257 (45)
- Higher education or university (%)	17 (11)	149 (26)
Current smokers (%)	128 (84)	218 (39)
History of hypertension (%)	35 (23)	35 (6)
History of hypercholesterolemia (%)	16 (11)	14 (3)
History of diabetes (%)	8 (5)	7 (1)
Family history of cardiovascular disease (%)	98 (66)	194 (36)
Birth weight-gram		
- Mean (SD)	3214 (676)	3370 (659)
- Median (range)	3150 (1500–5010)	3500 (1500–5800)
Body Mass Index – kg/m^2 ^– Mean (SD)	25.1 (0.4)	23.4 (0.2)
Waist circumference – cm (SD)	89.5 (13.1)	83.0 (10.2)
Waist/hip ratio (SD)	0.85 (0.006)	0.81 (0.008)
Premenopausal (%)	132 (87)	480 (85)

**Table 2:** Odds ratios (95%CI) for myocardial infarction in quintiles of birth weight as compared to the reference category

**Birth weight (g)**	**Odds Ratio (95% CI)**
> 3884	1*
3500–3883	1.3 (0.5–3.3)
3200–3499	1.4 (0.6–3.4)
3000–3199	1.7 (0.6–3.4)
< 3000	2.3 (1.0–5.4)

* Reference category

## Discussion

In this case control study we have found that women with a low birth weight had a higher risk of myocardial infarction than women with a higher birth weight. The data confirm the association found in cohort studies [13-17] and as such support the 'fetal origins of adult disease' or 'Barker hypothesis'.

A limitation of the study is that we have no information on gestational age. However, recently it has been demonstrated that both children who had been born prematurely and children who are small for gestational age had a reduction in insulin resistance [21,22]. The self-report of birth weights as well as the rather high percentage of missing values for birth weight may also limit the study, but the random events among cases and controls cannot explain our results. Socio-economic factors associated with low birth weight are also associated to risk factors for arterial disease later in life. As pointed out by several others, it will be nearly impossible to disentangle these effects [16,17].

In the present study we confirmed an interactive effect between low birth weight and a low educational level on the risk of myocardial infarction in women. However, when we adjusted for age, education level, body mass index, waist-hip ratio, hypertension, diabetes, hypercholesterolemia, smoking, and family history of cardiovascular disease, factors of which some may partly been seen as (the result of) socio-economic/environmental and genetic factors, we still observed an association between low birth weight and a higher risk of myocardial infarction. This is in agreement with others who also found that genetic and socio-economic circumstances at birth and in adult life can not completely explain the association between low birth weight and disease late in life [10,16,17,23-25].

Among the proposed underlying biological mechanisms to explain the association is impaired endothelial development. Already at very young age, individuals with low birth weight exhibit endothelial dysfunction that persists into childhood and adult life, suggesting that endothelial dysfunction precedes the development of vascular related diseases later in life and represents the link between low birth weight and these diseases [26-32]. Furthermore, Smith *et al*. [33] found that mothers, who once gave birth to thin babies, have a higher risk of developing ischemic heart disease later in life. Therefore, these mothers seem to have, just as their children, an impaired endothelial function. If the impaired endothelial function is already manifest during the process of implantation, it might lead to inadequate development of the vasculature in the maternal part of the placenta, which enfeebles the function of the placenta resulting in low birth weight.

## Conclusions

In conclusion, our study shows that a low birth weight (<2000 g) is associated with a 2.4 fold higher risk of myocardial infarction before the age of 50 as compared with a birth weight ≥ 2000 g. Because the risk of cardiovascular disease is known to increase with an increasing number of risk factors, women with a low birth weight should try to avoid acquired risk factors, like smoking and obesity. In addition, extra attention should be given in detecting diabetes or hypertension at a later stage in life and in detecting exaggerated growth during childhood, as these individuals seem most prone to develop disease later on in life [[34-36]].

## Competing interests

The author(s) declare that they have no competing interests.

## Authors' contributions

BT participated in the design, execution and analysis of the RATIO- study and drafted the manuscript.

KK analyzed the data and drafted the manuscript.

RH collected the data and performed statistical analyses.

FR initiated the study and helped to draft the manuscript.

FH participated in the design of the study and the writing of the paper.
